# Filtered circular fingerprints improve either prediction or runtime performance while retaining interpretability

**DOI:** 10.1186/s13321-016-0173-z

**Published:** 2016-10-31

**Authors:** Martin Gütlein, Stefan Kramer

**Affiliations:** Chair of Data Mining, Institute of Computer Science, Johannes Gutenberg - Universität Mainz, Staudingerweg 9, 55128 Mainz, Germany

**Keywords:** Fingerprints, (Q)SAR, Virtual screening, Feature selection

## Abstract

**Background:**

Even though circular fingerprints have been first introduced more than 50 years ago, they are still widely used for building highly predictive, state-of-the-art (Q)SAR models. Historically, these structural fragments were designed to search large molecular databases. Hence, to derive a compact representation, circular fingerprint fragments are often folded to comparatively short bit-strings. However, folding fingerprints introduces bit collisions, and therefore adds noise to the encoded structural information and removes its interpretability. Both representations, folded as well as unprocessed fingerprints, are often used for (Q)SAR modeling.

**Results:**

We show that it can be preferable to build (Q)SAR models with circular fingerprint fragments that have been filtered by supervised feature selection, instead of applying folded or all fragments. Compared to folded fingerprints, filtered fingerprints significantly increase predictive performance and remain unambiguous and interpretable. Compared to unprocessed fingerprints, filtered fingerprints reduce the computational effort and are a more compact and less redundant feature representation. Depending on the selected learning algorithm filtering yields about equally predictive (Q)SAR models. We demonstrate the suitability of filtered fingerprints for (Q)SAR modeling by presenting our freely available web service Collision-free Filtered Circular Fingerprints that provides rationales for predictions by highlighting important structural features in the query compound (see http://coffer.informatik.uni-mainz.de).

**Conclusions:**

Circular fingerprints are potent structural features that yield highly predictive models and encode interpretable structural information. However, to not lose interpretability, circular fingerprints should not be folded when building prediction models. Our experiments show that filtering is a suitable option to reduce the high computational effort when working with all fingerprint fragments. Additionally, our experiments suggest that the area under precision recall curve is a more sensible statistic for validating (Q)SAR models for virtual screening than the area under ROC or other measures for early recognition.

**Graphical Abstract:**

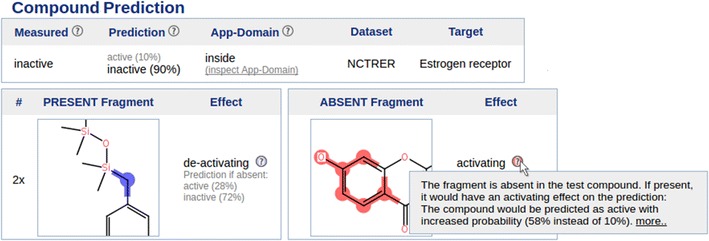

**Electronic supplementary material:**

The online version of this article (doi:10.1186/s13321-016-0173-z) contains supplementary material, which is available to authorized users.

## Background

(Q)SAR [(Quantitative) structure-activity relationship] models are effective tools to predict the biological or chemical activity of chemical compounds. The broad range of applications includes toxicity testing, where (Q)SAR modeling is an important alternative approach to lower the demand of in vivo animal testing. In drug design, lead compounds can be detected by applying (Q)SAR models for virtual screening, replacing extensive in vitro experiments [1]. The major advantage of the computer-driven approach is that these *in silico* models are faster and save money compared to conservative testing methods [2]. One of the drawbacks is that it is hard to understand the reasoning behind (Q)SAR model predictions: commonly (Q)SARs are built by training machine learning algorithms and therefore often resemble black boxes. However, interpretability of model predictions to discover a possible mode of action of the query compound is often demanded, e.g., by the OECD guidelines for valid (Q)SAR models [3]. A precondition for interpretable predictions is to train and apply the model with explicable and meaningful features. It is hard to extract any knowledge in case the feature space is encoded in a complex way and the chemical or biological information that the feature values are based on is opaque. One type of such encoded features are folded fingerprints.

A fingerprint is a bit-wise string, that includes only zeros and ones, encoding absences and presences of structural fragments. Often, fingerprints are folded to a fixed length to yield a compact representation of a potentially large list of structural fragments [4]. Folding introduces bit collisions, as the bit vector size is commonly much smaller than the number of features: multiple different fragments are assigned to the same position in a bit vector. The folding procedure is a one way transformation, i.e., it is not possible to distinguish between several structural features that have been mapped to a particular bit when having only the bit vector available. Bit collisions do not only remove interpretability, they also introduce ambiguity that could possibly deteriorate the prediction model. As an example, an important structural alert could be absent in a query compound. However, the query compound could possibly contain a completely different fragment that is assigned to the same bit position. Nevertheless, due to the sparsity of structural fragments (i.e., each fragment is usually missing in most of the compounds), folded fingerprints have been shown to work well in practice for building predictive (Q)SAR models (see below).

One category of structural fingerprints are the so-called circular fingerprints, which have already been introduced by Morgan in 1965 [5]. Despite their name, circular fingerprints yield single, separate structural fragments (that could possibly be employed without a bit-wise fingerprint). The fragments have a circular shape: starting with an initial atom, each fragment is extended by taking neighboring atoms into account. An important parameter for the fragments is the diameter that relates to the number of bonds. Fragments with diameter *zero* describe only the centering atom, fragments with diameter *two* contain all immediate neighbors of the center, diameter *four* includes a second layer of neighbors, and so on. A possible representation of circular fingerprint fragments assigns a numeric ID to each fragment, that is created by adding up the IDs of the atoms included in the fragment. The exact information about atoms encoded in the IDs depends on the fingerprint type. A common type is extended-connectivity fingerprints (ECFPs) [4], where the numeric IDs includes a range of atomic properties (like, e.g., atomic number, charge, valence, number of heavy atom neighbors, whether the atom is included in a ring, and so on). The chance that different fragments have the same numeric ID (without applying folding) has been shown to be slim.^1^ The fragments are mined separately for each molecule and can directly be mapped to the fingerprint bit vector by employing the numeric ID as hash-key. If folding is enabled, the numeric ID is reduced by multiples of the fixed bit vector length until it fits on the fingerprint (using the modulo operation). By default, fragments are enumerated without a support threshold (i.e., with a minimum frequency of 1).

Currently, there are two freely available cheminformatics libraries to mine ECFPs.^2^ Even though folded fingerprints are regularly applied for model building [6, 7], many researchers agree that bit-collisions might have a negative effect on modeling. Accordingly, researchers often use unprocessed fingerprints (i.e., without folding) to build (Q)SAR models [8–10]. A comparison, which was restricted to a single dataset, showed that unprocessed fragments can improve modeling results using Logistic Regression [11]. However, the same work stated the contrary for naive Bayes, unprocessed fingerprints for model building decreased the model performance. Additionally, in this study it was technically not feasible to build a random forest model due to the large amount of features. Rogers et al. [12] show that an adjusted version of naive Bayes can work well with unfolded fingerprints. Hence, naive Bayes is modified to employ only fragments that are present in the query compound for prediction and a correction term is introduced for infrequent features [13]. Also limited to a single dataset is the work of Liu et al. [14], who show that different folding sizes of 512, 1024, and 2048 produce only marginal model predictivity differences.

Circular fingerprints have initially been introduced for similarity searching. For this application, it has been shown that using unprocessed instead of folded fingerprints provides only a small performance gain [15, 16]. Hu et al. [17] present an approach that improves search results by employing only fragments that are present in active compounds.

We present, to the best of our knowledge, the first comprehensive and systematic comparison of unprocessed and folded fingerprints for (Q)SAR modeling and virtual screening. Moreover, we show that reducing the amount of features with endpoint-specific (i.e., supervised) feature selection is superior to folding. Filtering reduces the computational effort for modeling (similar to folding), while improving the model predictivity by avoiding bit collisions. Additionally, feature selection has the positive effect of retaining the interpretability of features. We demonstrate this by presenting the freely available modeling web service CoFFer (Collision-free Filtered Circular Fingerprints) that provides rationales for predictions.

This work is structured as follows. In the remainder of the “Background” section, we still elaborate on the type of validation conducted in the paper. In particular, we argue that AUPRC (the area under precision recall curve) is a suitable validation measure for virtual screening. The results section compares the performance of (Q)SAR models built with unprocessed, folded and filtered circular fingerprints and introduces our prediction web service. Details of the experimental setup and implementation are provided in the “Experimental” section. Subsequently, we provide a conclusion before presenting the methods used in this work.

### AUPRC (area under precision recall curve) as early recognition measure

A property of virtual screening datasets is the skewed class distribution: commonly, the number of active compounds is much lower than the number of inactives (in our context, also often referred to as decoys). Hence, accuracy is not a good option as predicting all or most compounds as inactive already yields a very high score.

The well known AUROC (the area under the receiver operating characteristic (ROC) curve) measure is based on ranking compounds according to their predicted probability of being active. *y*-axis and *x*-axis of the ROC curve are true positive rate (also named sensitivity or recall) and false positive rate [18]. The area under the ROC curve usually ranges between 0.5 (random) and 1.0 (perfect), and has the nice property that it can be interpreted as the probability that a randomly drawn positive instance is ranked higher than a randomly drawn negative instance. However, AUROC has the drawback that all compounds within the ranking have equal weight, whereas in virtual screening, researchers are usually more interested in the compounds that are most likely active, and less interested in the compounds that are less likely active. Hence, early recognition measures like EF (enrichment factor) and BEDROC (Boltzmann-Enhanced Discrimination of ROC) have been developed [19, 20].

Enrichment factor (EF) compares the ratio of active compounds in the entire dataset to the ratio of active compounds within the top $\chi$ ranked compounds [20]. The researcher has to manually decide on a threshold $\chi$ (often one or five percent is selected). EF is insensitive to changes that do not “cross” this threshold (e.g., the enrichment factor does not increase when the ranking improves within the top $\chi$ percent). Moreover, the score is based on the original ratio of active compounds in the entire dataset and can hardly be used to compare predictions on datasets with different class distributions.

BEDROC is based on robust initial enhancement (RIE), which uses continuously decreasing exponential weight when ranking compounds according to estimated probability [19]. It is bounded between 0 and 1. BEDROC has the drawback to depend on a parameter *alpha* that defines the exponential weight (and therefore its sensibility towards early recognition).

We here propose to use the area under the precision recall curve (AUPRC) as validation statistic. To the best of our knowledge it has not been used in virtual screening so far, even though it has been described as a more sensible measure on datasets with skewed class distributions [21–23] than the area under the ROC curve (AUROC). Moreover Davis et al. [21] show that, when comparing validation results, AUROC dominates if and only if AUPRC dominates and that algorithms that optimize AUROC not necessarily optimize AUPRC. Similarly to the ROC curve, the precision recall curve has the true positive rate (recall) as *y*-axis, however on the *x*-axis precision is employed (also referred to as selectivity or positive predictive values). For high probability values the curve is calculated with only few compounds as all true negative predictions are ignored by precision and recall. To this end, predictions with high probability have a higher influence on AUPRC than predictions with lower probability, which is desirable when analyzing virtual screening results.

As a drawback, the baseline of the area under the precision recall curve is equal to the ratio of active compounds in the dataset. In other words, the AUPRC score ranges between the ratio of active compounds (random prediction) and 1 (perfect prediction). This dependency of AUPRC hinders comparisons of margins of improvement between datasets with different class distributions and reduces the interpretability of AUPRC scores. Accordingly, the precision under the precision recall curve is neither “independent to extensive variables” [24] nor is its interpretation as intuitive as, e.g., AUROC scores. However, it full-fills other favorable characteristics of validation measures listed by Nicholls [24] as it has no free parameters, and can be estimated in a robust way providing confidence intervals [25]. Overall, we consider AUPRC to be an appropriate measure for validating virtual screening experiments especially due to its suitability for skewed class distributions.

In Table 1, we give an artificial prediction example that demonstrates the above described properties of AUROC, EF, and BEDROC and outlines why AUPRC may be preferable in virtual screening.

**Table 1:** Comparing AUPRC to other virtual screening measures when improving a reference ranking (a) at different positions (b–e)

Ranking of active (1) and inactive (0) compounds due to predicted probability										
(a)	11011111110111000000010000000000000000000000010000000000000000100									
(b)	11011111110111000000010000000000000000000000010000000000001000000									
(c)	11011111110111000000010000000000000000000100000000000000000000100									
(d)	11011111110111000100000000000000000000000000010000000000000000100									
(e)	11111101110111000000010000000000000000000000010000000000000000100									

	AUROC	$\Delta$	AUPRC	$\Delta$	EF-5%	$\Delta$	BEDROC-20	$\Delta$	BEDROC-100	$\Delta$
(a)	0.864		0.772		2.9		0.884		0.885	
(b)	0.869	+0.005	0.773	+0.001	2.9	0	0.889	+0.005	0.889	+0.003
(c)	0.869	+0.005	0.774	+0.002	2.9	0	0.889	+0.005	0.89	+0.004
(d)	0.869	+0.005	0.78	+0.009	2.9	0	0.89	+0.006	0.892	+0.006
(e)	0.869	+0.005	0.822	+0.051	4.3	+1.4	0.89	+0.006	0.893	+0.008

Each bit resembles an active (1) or inactive compound (0). Each bit-string *(a–e)* corresponds to the result of a classifier that ranks all test-set compounds according to their predicted probability of being active. A perfect prediction would list all “1s” before “0s”. The reference prediction *(a)* has an AUROC (the area under the ROC curve) score of 0.864 (Hence, the probability that a randomly drawn active compound is ranked higher than an inactive compound is 86.4%). We improve the reference prediction *(a)* by modifying the predicted probability of a single compound at different positions in the ranking: in *(b–d)*, a single active compound is predicted with higher probability and shifted 4 positions upwards in the ranking. In *(e)*, a single inactive compound is predicted with lower probability and is moved 4 positions downwards in the ranking. When performing virtual screening, the last change *(e)* is probably most important to us, as we are interested in the compounds that are most likely active. However, the change in AUROC is constant for *(b–e)*. EF (enrichment factor) and BEDROC (Boltzmann-Enhanced Discrimination of ROC) have the disadvantage of relying on a user defined parameter. Moreover, EF-5% changes only if the number (not the ordering) of active compounds within the top $\chi$ bits (here: 3) differs. On the contrary, AUPRC (the area under precision recall curve) has the desired property that it increases more when the affected ranking position is higher without relying on a parameter (Please find ROC and precision recall curves for *(a–e)* in the Additional file 1)

## Results and discussion

This section is divided into two parts. The first part compares model building results for folded, unprocessed, or filtered circular fingerprint fragments. The second part presents a freely available (Q)SAR prediction service that provides a rationale for each prediction based on un-folded, interpretable circular fingerprints.

### Comparison of folded, unprocessed, and filtered circular fingerprints

The main result of this work is summarized in Table 2. Modeling with folded fingerprints is fast but removes interpretability and decreases the performance of prediction models. Employing unprocessed fingerprints (i.e., not restricting the number of features by folding) mostly yields better models with unambiguous features, while being slower due the increased number of features. Alternatively to folding, we apply supervised feature selection (as described in the “Methods” section) to limit the amount of fragments. Filtered fingerprints are fast, retain interpretability, and produce models with competitive predictivity (depending on the selected algorithm).

**Table 2:** Overview of results

Selection of fragments	Intepretable fragments	Fast processing (low num. features)	Best performance		
			RF	SVM	NB
Unprocessed	Yes	–	Yes	Yes	–
Folded	–	Yes	–	–	–
Filtered	Yes	Yes	Yes	–	Yes

*Unprocessed* fragments yield random forest (RF) models and support vector machine (SVM) models with good performance and retain interpretability, but require a high computational cost. *Folded* fragments allow fast processing, but generate inferior models and are non-interpretable due to bit collisions. *Filtered* fragments yield the best naive Bayes (NB) models and can be employed to build RF models that are equally good as those built with unprocessed fragments. Filtered fragments also retain interpretability and allow fast processing

In summary, unprocessed (all) fragments are a good option if there are enough computational resources to optimize SVMs and the vast amount of (often redundant) features does not hinder interpreting predictions. Otherwise, filtered fragments should be preferred

In general, RF models yield good results without parameter tuning, however, SVM models are usually better when their parameters have been optimized (see section on parameter optimization)

#### Initial results with default parameters

In this work, we have selected three well known machine learning algorithms: random forests, support vector machines, and naive Bayes. The algorithms are applied to a range of 76 datasets (see “Experimental” section). Initially, we have selected ECFPs (extended-connectivity fingerprints) with diameter four. The size of the folded fingerprint is 1024, which is the probably most commonly used bit-vector length. To ensure a fair comparison, we have applied our supervised filtering method to select 1024 features as well.

**Fig. 1:**
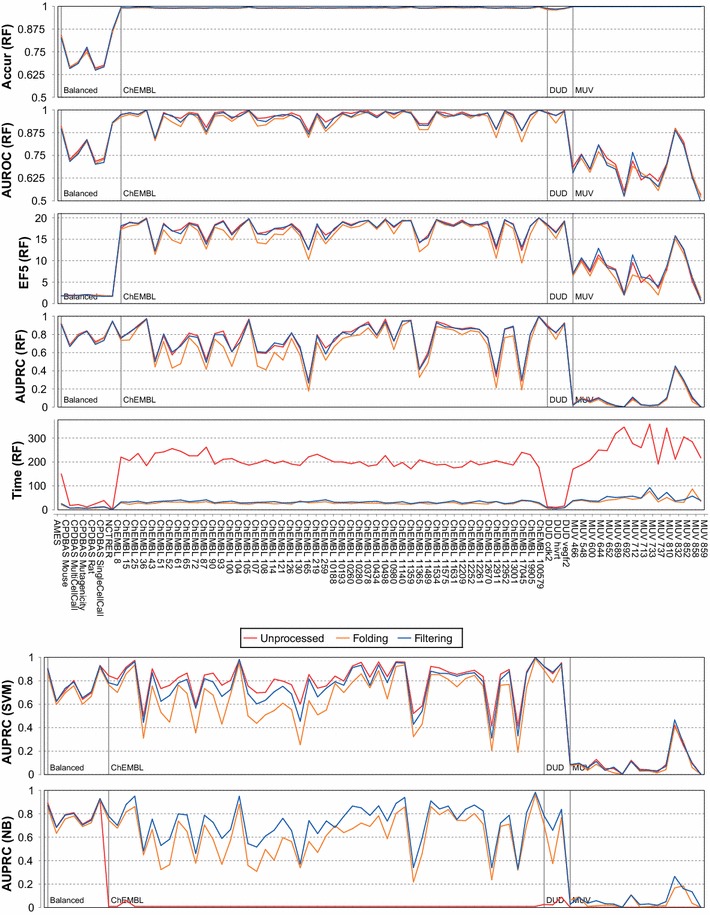
Validation results for ECFP4 for unprocessed, folded and filtered fingerprints (folded/filtered bit-vector size is 1024). For the random forest (RF) algorithm, 5 measures are provided for each of the 76 datasets. The difference between folded fingerprint features and filtered/unprocessed features is less distinct considering the area under the ROC curve (AUROC), and enrichment factor (EF), and more distinct considering the area under precision recall curve (AUPRC). Run-time measure the seconds to mine fragments and build a model and is highest for unprocessed features. The remaining charts for support vector machines (SVM) and naive Bayes (NB) are provided in Additional file 2

Figure 1 shows accuracy, the area under the ROC curve, enrichment factor, the area under the precision recall curve and the run-time for random forests. As already outlined before, accuracy is not a suitable validation measure for highly unbalanced virtual screening datasets. AUROC is very similar for the three feature types, even though it can be seen that folding is slightly worse than the other two feature types. This distinction is more evident for EF, and even more distinct when comparing results for the area under precision recall curves. The run-time required for mining features and training a model is multiple times higher if unprocessed fingerprints are used. Figure 1 also provides AUPRC scores for support vector machine models, which are higher when applying all fragments compared to filtered fragments (folding is clearly worst). This is due to the low number of selected features (1024), as shown below. Folded fragments produce support vector machines with the lowest predictivity. The bottom chart of Fig. 1 shows a different result for naive Bayes. Here, the best models can be created with filtered fragments, whereas unprocessed fragments create the worst models. As already indicated in the introduction, naive Bayes cannot cope with many sparse and redundant features.

**Fig. 2:**
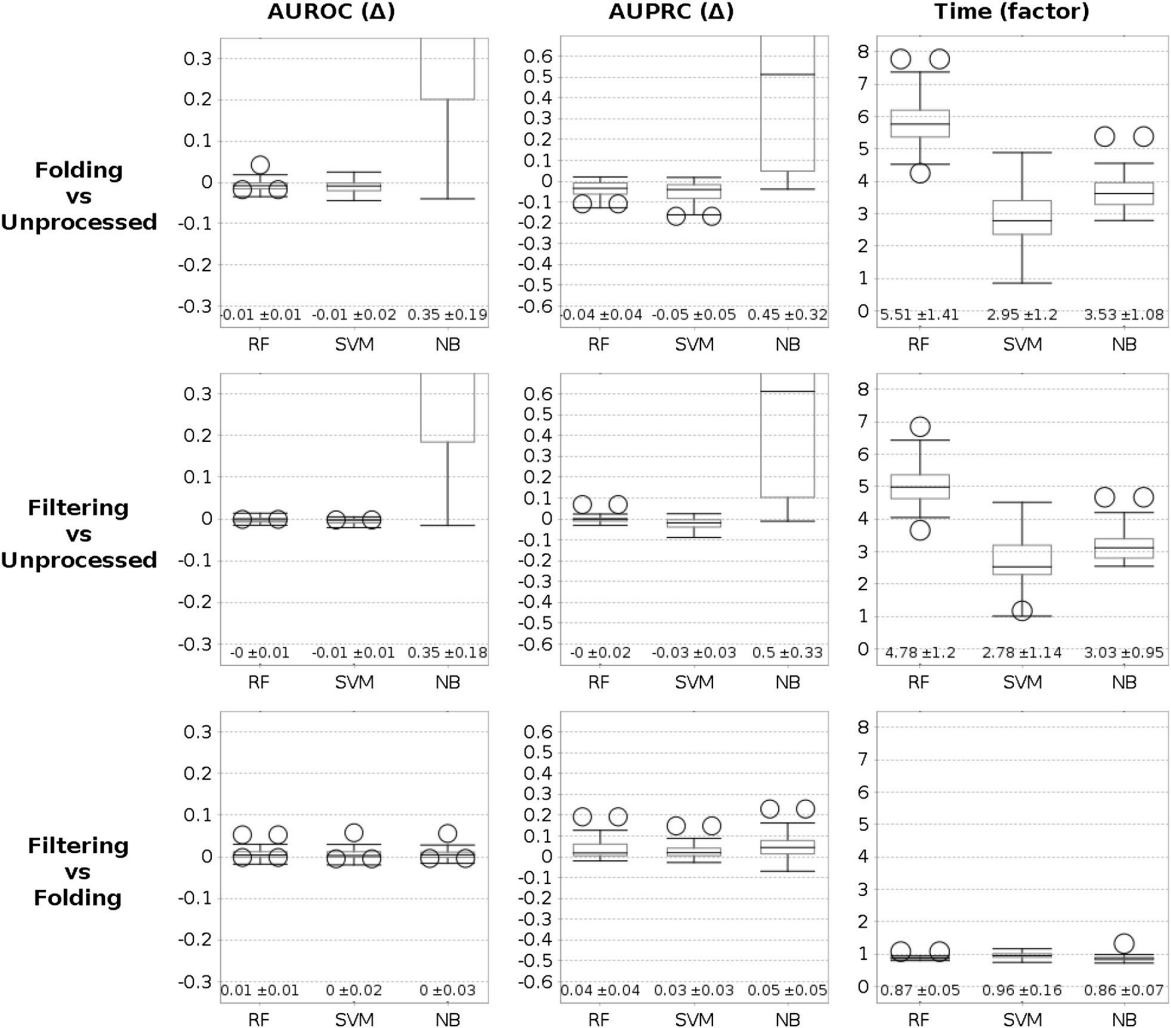
Mean changes in AUROC and AUPRC when comparing filtered, folded and unprocessed fingerprints (ECFP4 with bit-vector size 2048). The applied machine learning algorithms are random forests (RF), support vector machines (SVM) and naive Bayes (NB). Note that the scale for the area under the ROC curve (AUROC) is half the scale of the area under prediction recall curve (AUPRC): both measures have a maximum value of 1, however, the baseline of AUROC scores is 0.5 whereas the baseline of AUPRC is close to 0 on some datasets (on the MUV datasets, the ratio of active compounds is 0.002). Run-time corresponds to the speedup for mining the features and building a model of filtering/folding compared to unprocessed fragments. The run-time of filtering is only slightly slower than folding, as both methods yield an equal number of features and the actual filtering routine is fast (on average 5.1 s) compared to the entire model building process (38.4 s for RF)

For the experimental results shown in Fig. 2 we increase the bit-vector length from 1024 to 2048. The figure shows the average margin of improvement or degradation in AUROC, AUPRC and model building run-time. Again, the area under the precision recall curve is more sensible than the area under the ROC curve. Applying folded instead of unprocessed fingerprints reduces predictivity of random forest and support vector machine models ($\Delta$ AUPRC: $-0.04$ and $-0.05$), whereas naive Bayes profits from the more compact feature representation ($\Delta$ AUPRC: $+0.45$). Building models with folded fingerprints is up to 5.51 times faster. Filtering yields better results than folding for all algorithms ($\Delta$ AUPRC: ranging from $+0.03$ to $+0.05$). When comparing filtering to unprocessed fragments, random forest models have on average equal performance, naive Bayes models are much more predictive ($\Delta$ AUPRC: $+0.5$), whereas support vector machines are slightly worse ($\Delta$ AUPRC: $-0.03$). In general, employing the area under the ROC curve instead of the area under the precision recall curve for the comparison leads to equal trends, yet smaller differences.

#### Modifying the number of selected features, the diameter and the fingerprint type

The number of bit collisions introduced by folding decreases with rising bit-vector size, as shown in Table 3. Moreover, the number of unprocessed fragments increases with increased diameter of the circular fragments: encoding only single atoms (diameter zero) yields on average 57 fragments on our datasets, whereas about 80 thousand fragments are found with diameter six.

**Table 3:** Average number of fragments and bit-collisions when folding circular fingerprints on our datasets

Type	Fragments	1024		2048		4096		8192	
		Rate	Bit-load	Rate	Bit-load	Rate	Bit-load	Rate	Bit-load
ecfp6	80,342.54	1	78.46	0.99	39.24	0.98	19.64	0.95	9.86
ecfp4	23,874.58	0.99	23.32	0.98	11.68	0.94	5.89	0.8	3.11
ecfp2	2169.37	0.7	2.39						
ecfp0	57.01								

*Rate* is the ratio of bit positions that are mapped by more than one fragment (e.g., 99% of bit-positions correspond to multiple fragments for ECFP4 and bit-vector size 1024). *Bit-load* is the mean number of fragments that are mapped to a single bit

**Fig. 3:**
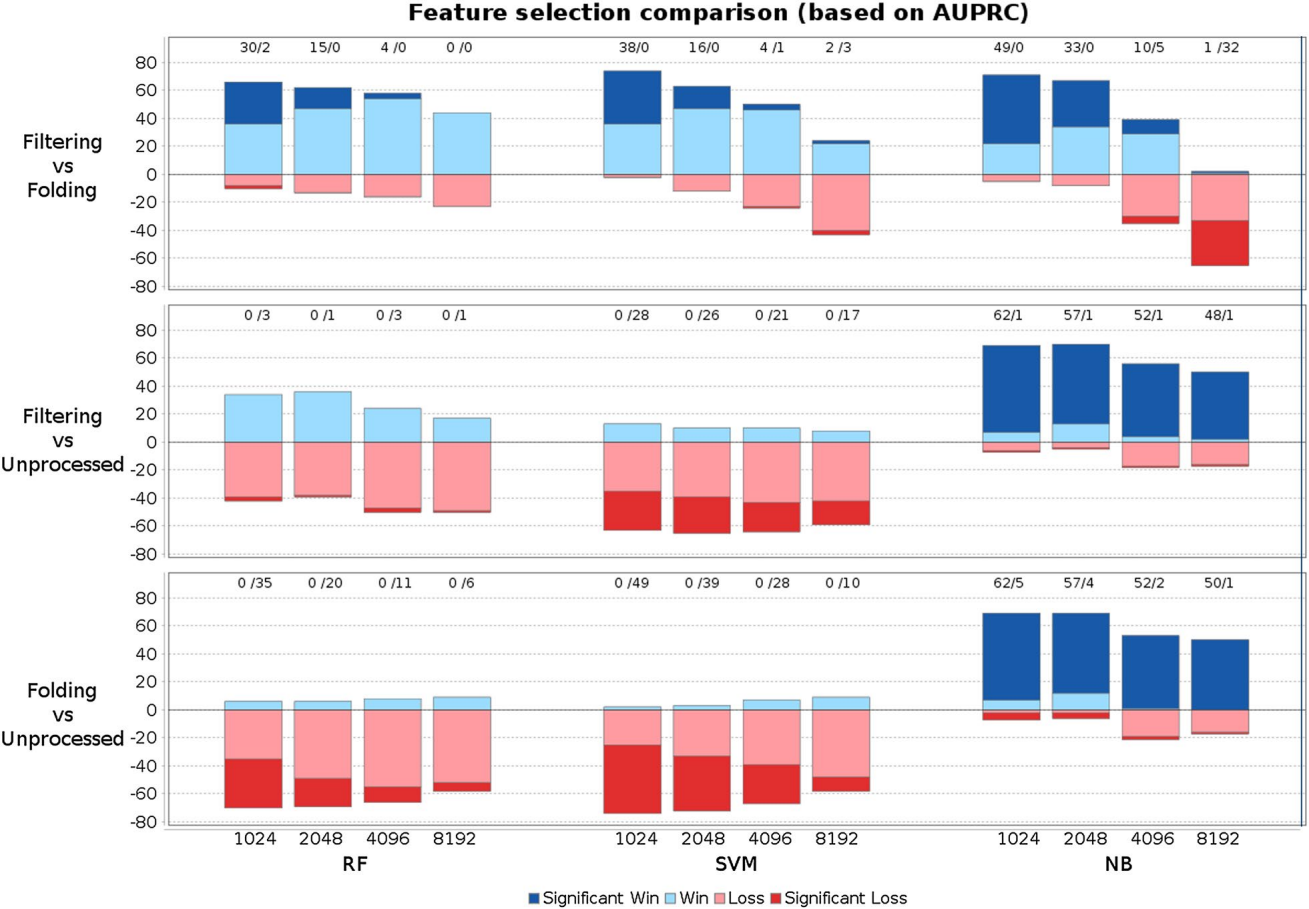
Win loss statistics to compare AUPRC scores of different feature selection techniques with increasing bit-vector lengths (using ECFP4). *Each bar* corresponds to a pairwise comparison of two methods on 76 datasets. Wins of the first/second method are colored in *blue*/*red colors* and drawn above/below zero respectively. *Intense colors* indicate significant wins/losses and are additionally stated in numbers above *each bar*. When comparing filtering/folding to unprocessed fragments, increasing bit-vectors for filtering/folding are compared to the entire unprocessed feature set. Filtering is in general better than folding. The longer the bit-vector, the more similar are the folded or filtered feature sets to the unprocessed feature set. Filtered features yield equally predictive models as unprocessed fragments for RF, less predictive models for SVM and largely improved models for NB. Folding deteriorates predictivity compared to unprocessed fragments unless NB is used

Figure 3 presents modeling results with variable bit-vector size for ECFP4, by providing win-loss statistics for AUPRC for all 76 datasets. Obviously, the higher the number of features, the lower is the difference between unprocessed features and filtered or folded fragments. The superiority of filtering over folding is especially evident for small bit-vector lengths. For random forests and support vector machines, applying unprocessed features works very well. For random forests, filtering yields about equally good results when using e.g. 2048 filtered compared to unfiltered fragments (36 wins and 39 losses, 1 loss significant). Support vector machines are best when trained with unprocessed fragments, e.g., the degradation is significant for 26 datasets when using 2048 filtered features compared to all, unprocessed features. Naive Bayes fails to build predictive models with unprocessed (sparse) features. Hence, folding improves its performance (compared to unprocessed fingerprints). However, filtering with bit-vector length 1024 yields the overall best models naive Bayes models in this setup.

**Fig. 4:**
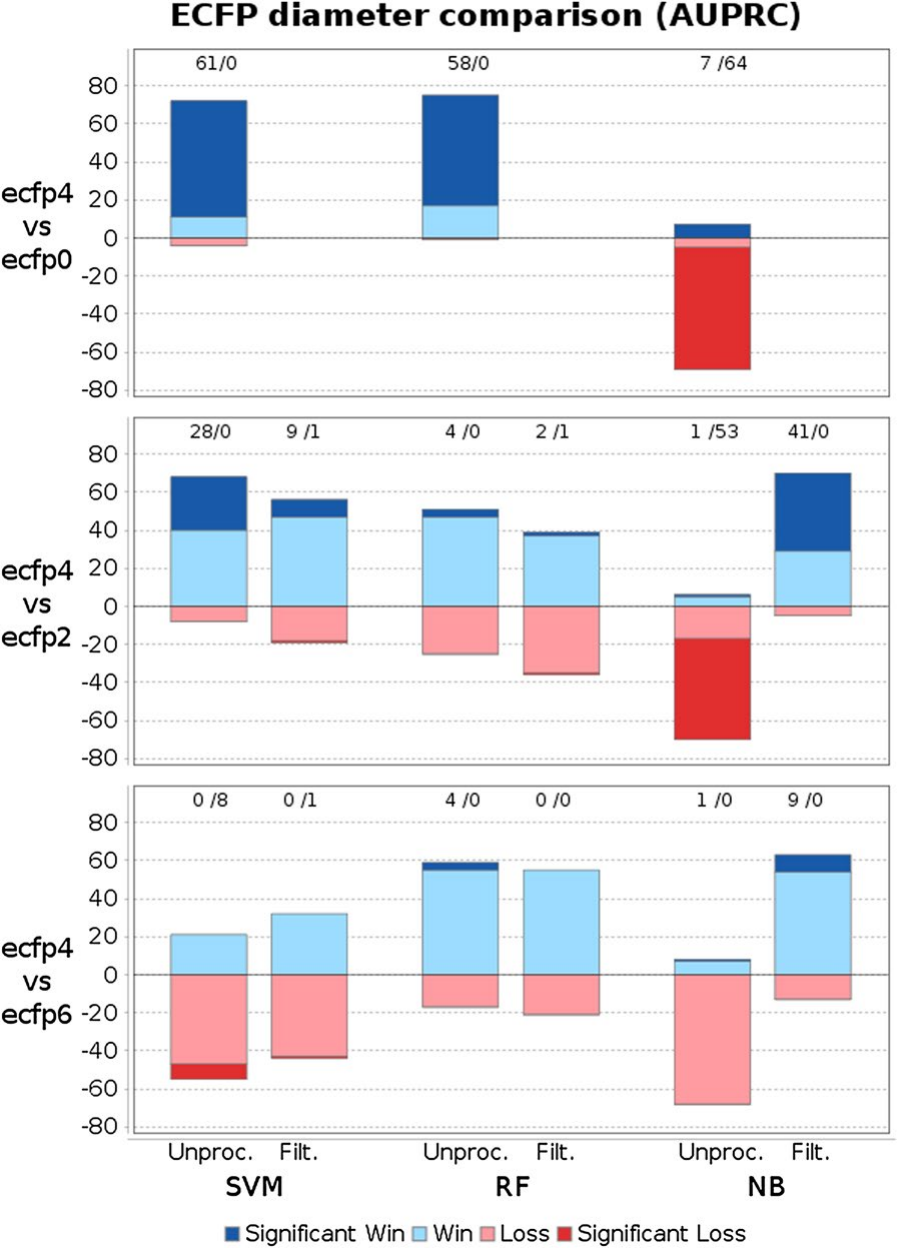
Win-loss statistics to compare AUPRC scores of ECFP diameter 4 to diameters 0, 2, and 6 (for unprocessed fingerprints and 1024 filtered fingerprint fragments). *Each bar* corresponds to a pairwise comparison of two methods on 76 datasets. Wins of the first/second method are colored in *blue*/*red colors* and drawn above/below zero respectively. *Intense colors* indicate significant wins/losses and are additionally stated in numbers above *each bar*. Diameter 4 yields in general the most predictive models. Exceptions are naive Bayes and diameter 0 and 2, which is due to the low number of features with low diameter. However, when applying filtering ECFP4 works best for naive Bayes. Moreover, slightly more predictive support vector machines can be build using ECFP6

**Fig. 5:**
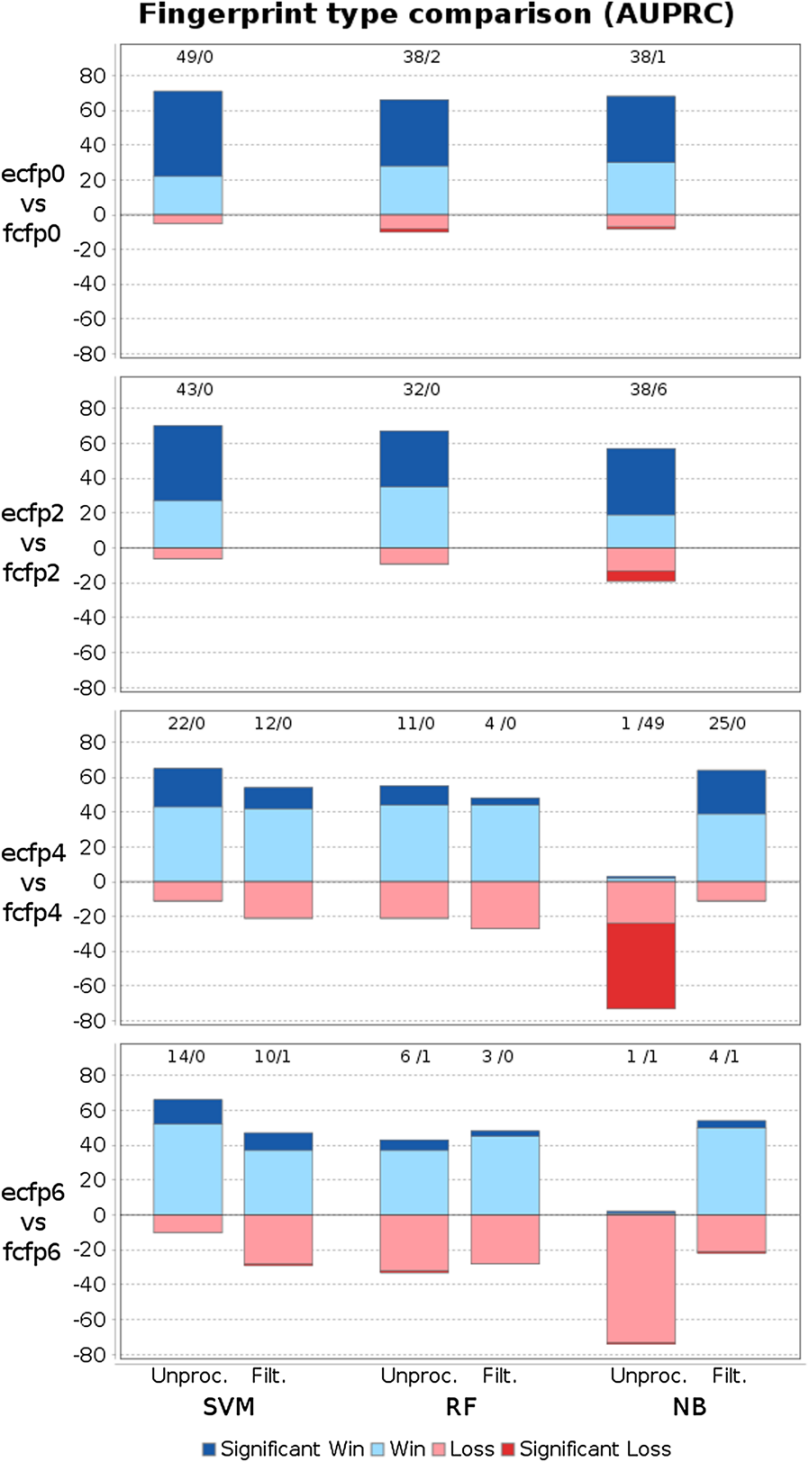
Win-loss statistics comparing ECFPs vs FCFPs at different diameters for unprocessed and filtered (1024) fragments. *Each bar* corresponds to a pairwise comparison of two methods on 76 datasets. Wins of the first/second method are colored in *blue*/*red colors* and drawn above/below zero respectively. *Intense colors* indicate significant wins/losses and are additionally stated in numbers above *each bar*. ECFPs are mostly better when using unprocessed features, except naive Bayes for diameters 4 and 6. The explanation is the lower number of features produced by FCFPs. When applying filtering, ECFPs always lead to more predictive models than FCFPs

Moreover, we compare ECFP diameter 4 to diameters 0, 2, and 6 using raw features and filtering with size 1024 (in Fig. 4). Even though the best diameter setting depends on the particular dataset, diameter 4 works in general best for random forests and naive Bayes, whereas diameter 6 works slightly better for SVMs. Functional class fingerprints (FCFPs) are a variant of ECFPs that are less precise and describe substructures according to their role in pharmacophores. Accordingly, FCFPs produce less features and therefore less bit collisions (see supplementary file). However, ECFPs work in general better for model building, as shown in Fig. 5.

#### Parameter optimization for each dataset

In order to train a highly predictive model for each dataset, we optimize the features and model algorithms including their parameters. To not over-fit the dataset and to obtain a realistic predictivity estimate, we add a nested level of cross-validation [26, 27]. Detailed information is given in the “Experimental” section. On most datasets, the best model could be built by optimizing support vector machines (see Table 4). The predictivity estimates for our final models can be found in the supplementary material. Our models compare well to published work on the same datasets. Regarding the subset of 7 balanced datasets, our approach outperforms existing studies [28–31] or yields equally predictive models [8]. The latter work also trained highly optimized support vector machines on the 16 MUV (Maximum unbiased validation) datasets and produced models with similar AUROC to our models (7 wins, 8 losses, 1 draw). Additionally, we examined results of a consensus modeling study [6] that was applied to all 69 virtual screening datasets used in that work. (All datasets are included in a bench-marking platform provided by the same authors [16]). However, completely differing validation techniques^3^ render a comparison impossible and our approach yielded a higher area under the ROC curve in 66 out of 69 cases. Please also refer to the supplementary material for details on the comparison to the other methods.

**Table 4:** Number of selected configurations for optimized web service models (for each of the 76 datasets)

Number of features	1024	2048	4096	8192
Times selected	24	17	10	25
Algorithm	RF	SVM	NB	
Times selected	14	60	2	

To limit the number of parameters we skip FCFPs and set the ECFP diameter to 4. For support vector machines different parameters have been optimized (as described in ”Experimental” section). A complete list of selected parameters and nested cross-validation results can be found in Additional file 2

### CoFFer—a prediction web service with interpretable predictions

We created a prediction web service to demonstrate the feasibility and utility of the previously described filtering approach. The web service is called CoFFer (Collision-free Filtered Circular Fingerprints). It is open-source. A freely available prototype is running at http://coffer.informatik.uni-mainz.de. It offers 76 (Q)SAR models that can be applied to untested query compounds. The service ranks and highlights the circular fingerprint features that have been used by the (Q)SAR model to help interpreting the prediction result.

**Fig. 6:**
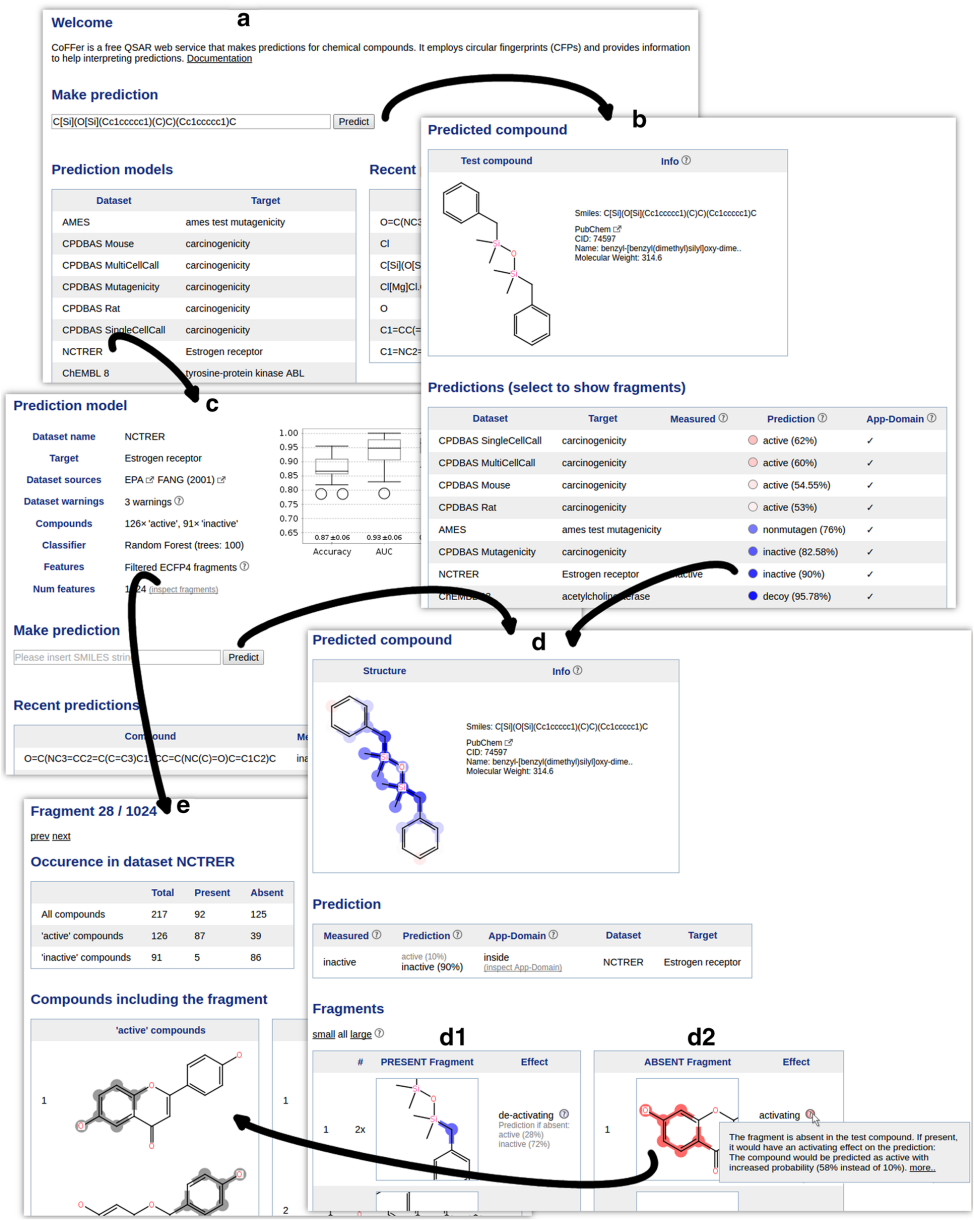
Workflow of the freely available CoFFer prediction web service. The service is available at http://coffer.informatik.uni-mainz.de. Please refer to the results section for a description

Figure 6 assembles screen-shots of the web service to outline the work flow. The entry page *(A)* lists the available (Q)SAR models and allows predicting a compound (provided as SMILES) for all endpoints simultaneously *(A* $\rightarrow$ *B)*. Alternatively, the researcher can handpick a model, to inspect its properties *(A* $\rightarrow$ *C)* before predicting a query compound *(C* $\rightarrow$ *D)*. The information given alongside a model prediction includes two lists of fragments, containing present and absent fragments within the query compound *(D1+D2)*. We have developed a ranking scheme for fragments to show fragments at the top that have the highest influence on the prediction. Fragments that have a *deactivating* effect on the prediction are colored in blue, *activating* fragments are colored in red. Additionally, atoms and bonds of the query compound are colored by summarizing the effect of single fragments (which are present in the query compound). Ranking and highlighting methods are described in the “Methods” section. Additionally, each model determines whether the query compound belongs into its applicability domain [32] *(B, D)*. We employ a distance-based approach that rejects a query compound if the Tanimoto distance to its three most similar training dataset structures is too high.^4^

The example prediction provided in Fig. 6 employs a random forest model that was built on the NCTRER dataset, hence, it predicts whether a compound binds to the estrogen receptor. The predicted compound is already included in the training dataset, as indicated on the prediction result page *(D)*. The top ranked absent fragment that is shown by our service is a pattern matching a phenolic ring *(D2)*. The service marks this fragment as activating, stating that this compound would be classified as active if the compound would match this fragment. This resembles findings by Fang et al. [33] that have developed a rule set for the identification of estrogen receptor ligands. They state that a phenolic ring is an important precondition for a ligand. Moreover, Fang et al. outline that a rigid structure is important for a successful binding. Similarly, our highest ranked present fragment is an aliphatic carbon with two single bonds *(D1)*, a fragment that occurs twice in this compound and makes it very flexible. Our service renders this fragment as deactivating, as the query compound would be predicted as active with increased probability if this fragment would not match the query compound.

Finally, the CoFFer web service provides information about the occurrence of the employed fingerprint features in the training dataset *(E)*. Here, the phenolic ring pattern matches 92 compounds, 87 of these compounds are estrogen receptor ligands.

## Experimental

### Datasets

The 76 datasets selected for this study are listed in Table 5. They include 69 benchmark datasets for virtual screening provided by Riniker et al. [16]. To enlarge and widen the range of datasets, we added 7 balanced datasets (i.e., datasets with about equal amount of active and inactive class values).

**Table 5:** The 76 datasets used for our model building experiments

Type	Dataset/group	Num	Compounds	Active	In-active	Source
Balanced	AMES	1	4337	2401	1936	[47]
Balanced	CPDBAS	5	1102.6	545.8	556.8	[48]
Balanced	NCTRER	1	217	126	91	[33]
Virtual-screening	ChEMBL	50	10,100	100	10,000	[6, 49]
Virtual-screening	DUD	3	1822.3	42	1780.3	[6, 50]
Virtual-screening	MUV	16	15,026.8	30	14,996.8	[6, 51]

Multiple occurrences of the same compound are inserted only once. E.g., some of the originally 15,000 decoys for each MUV dataset are removed. In case, multiple occurrences have differing endpoint values, the compound is omitted. Only 5 of 7 endpoints from the CPDBAS dataset could be used for this study as two endpoints (Hamster and Dog/Primates) are to small and yield less than 1024 ECFP4 fragments. A more detailed list of datasets is provided in Additional file 2

### Algorithms and validation

We have used the machine learning library WEKA [34] (v3.7.13) for modeling and selected three well known classifiers: random forests [35], naive Bayes [36] and support vector machines [37]. A 3-times repeated tenfold cross-validation has been applied to compare folded, filtered and unprocessed features sets (employing default algorithm parameters without optimization). Filtering of fragments has been carried out within the training data fold of each cross-validation to avoid information leakage. Significance tests have been performed with a corrected paired t test with a *p* value threshold of 0.05 [38].

To build the most predictive model for each dataset, we optimized support vector machine parameters and selected the best number of features (see Fig. 4). In order to avoid estimating an over-optimistic validation score, we applied a nested $3 \times 3 \times 10$-fold cross-validation. Model selection is performed on the inner cross-validation loop, and the predictivity estimate of the selected model is evaluated with the outer cross-validation loop. To limit the huge computational effort of nested cross-validation, we fixed fingerprint type and diameter to ECFP4. Within the inner loop of the nested cross-validation we selected the number of fingerprint fragments (1024–8192) and the best parameters for support vector machines. We tested *c*-values 1, 10, and 100 with a linear kernel and a radial basis function (RBF) kernel. Additionally, gamma values 0.001, 0.01 and 0.1 are tested for the RBF kernel. Random forests and naive Bayes have been applied with default parameters (which is 100 trees for the random forest algorithm). See Additional file 2 for detailed results. The HPC cluster of Johannes Gutenberg Universität Mainz, Mogon, made this computationally extensive evaluation feasible.

### Implementation

This work has been implemented in the Java programming language. It is divided into four packages that are available as open-source libraries on GitHub (see https://github.com/kramerlab). Moreover, the libraries are organized as maven package and can easily be integrated into other packages.

**cdk-lib**: provides mining and filtering of circular fingerprints. It is based on the implementation of circular fingerprints in the chemistry development kit (CDK) [39]. Our library adds different bit-vector lengths as well as our filtering approach. Moreover, we provide a depiction functionality to draw a circular fingerprint fragment within a compound.
**weka-lib**: extends the machine learning framework WEKA with a nested cross-validation and basic ranking functionalities to sort features according to their influence on a prediction.
**cfp-miner**: is based on the previous libraries and allows to build and validate (Q)SAR models with circular fingerprint fragments.
**coffer**: provides the Apache CXF implementation of the CoFFer web service (currently running here: http://coffer.informatik.uni-mainz.de). Additionally to the graphical user interface, it has a REST interface that simplifies the integration of our service into other frameworks and is compliant with the OpenTox API [40]. Moreover, our web service accesses PubChem and ChEMBL to provide additional information for the predicted compounds, and maintains links to sources of modeled endpoints and datasets.

## Conclusions

Circular fingerprints can be applied to yield highly predictive (Q)SAR models. Commonly, either unprocessed fingerprints or folded fingerprint fragments are employed, even though the latter introduces bit collisions. This work provides a comprehensive comparison between folded and unprocessed fingerprints. We show that folding improves the model building run-time but yields slightly (yet often significant) less predictive models. Unprocessed fingerprints have also the advantage of retaining interpretability of structural fragments. We introduce a supervised filtering approach, that combines the advantages of both methods: it produces a smaller, less redundant set of interpretable structural features, reduces the computational effort for model building, and yields predictive models. For the presented validation study, we selected the area under precision recall curve (AUPRC) as validation measure. This statistical measure is preferable to the commonly used area under ROC, as it has the advantage of being more sensible to predictions of compounds that are predicted as active with high probability. Moreover, we present a prediction web service that showcases our approach and provides rationales for predictions. To this end, we developed a technique to rank the structural fragments according to their influence on the prediction. The model service is open-source, freely available and can be accessed directly with the browser or with a REST interface.

In the future, we plan to integrate the prediction models into the OpenTox service ToxPredict [40]. Additionally, filtered circular fingerprints could be incorporated into our 3D-space mapping and visualization tool CheS-Mapper [41].

## Methods

### Filtering fingerprints with supervised feature selection

We apply supervised feature selection to limit the amount of circular fingerprint fragments as an alternative to folding. The main advantage of this method is that bit-collisions are avoided. Hence, well-defined structural fragments that are either present or absent in a query compound are employed as features for the (Q)SAR model. The input for our method is a list of fragments (x-values) and the endpoint (y-values) of the training set compounds. The output is a reduced number of x-values, i.e., the number of columns in the training dataset decreases.

The high-level pseudo-code for our method is given in Table 6. After stripping compounds that match only a single compound, we remove non-closed fragments. In the context of graph mining, a fragment is not closed if there exists a sub-fragment that matches the exact same set of compounds. Closed fragment mining has been shown to be an effective way to greatly reduce the number of features and decrease redundancy [42]. Subsequently, if the number of fragments is still to large, supervised feature selection is applied using a $\chi ^2$ filter. Hence, features that have no measurable correlation to the target endpoint are removed. This supervised feature selection method for molecular fragments has been successfully applied (and is nicely explained) by Maunz et al. [43]. The implementation of our filter method for circular fingerprints is freely available as described in the “Experimental” section above.

**Table 6:**
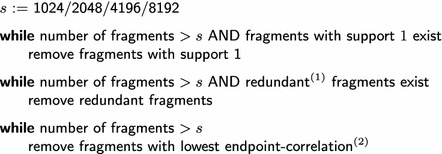
Pseudo-code for filtering circular fingerprints with supervised feature selection

$^{(1)}$Redundant := non-closed; i.e., a fragment is redundant if there exists a sub-fragment that matches the same compounds.

$^{(2)}$Fragments with the lowest *p* value of a $\chi ^2$ test are removed; the test measures the correlation between the endpoint value distribution of all compounds, and the compounds that match the fragment

### Ranking of structural fragments that are used as (Q)SAR prediction features

Our method ranks features according to their contribution to a particular (Q)SAR model prediction. Subsequently, the most important present and absent substructures in the query compound can be presented to the user and aid in understanding the (Q)SAR model and in deriving a mechanistic interpretation. The input for our method is a classifier for binary class values (e.g. active and inactive), a query compound, and the structural features to be ranked. The result of our method is a ranking of features. Additionally, the features are divided into two lists of fragments that are either present or absent in the query compound. Moreover, it is determined whether a feature has an activating or deactivating effect on the prediction. An example is given in the result section [see *(D1)* and *(D2)* in Fig. 6].

The importance of a feature is computed by swapping the feature value of the fragment and re-classifying the compound. The features are ranked according to the absolute value of the difference in predicted probability. The prefix of the change is used to tag the feature as activating or deactivating: a feature is marked as activating if it is originally present in the compound and a re-classification with swapped feature value leads to a lower probability of being active. Also, a feature is marked as activating if it was originally absent in the query compound and the predicted probability with swapped feature value leads to a higher probability to be active. Otherwise, we consider the feature to be deactivating.

When swapping feature values for a fragment, the method takes the compound structure into account. If the evaluated fragment is originally present in a compound, its super-fragments (that extend this fragment) will be switched off as well when evaluating the importance of the fragment. Additionally, sub-fragments that are included in this fragment and do not match the compound at a different location are disabled. Accordingly, if the evaluated fragment is originally absent in the compound and is switched on for evaluation, then all sub-fragments (that are contained within this fragment) are switched on simultaneously.

A drawback of this method is that it might be computationally extensive for large feature sets and slow prediction algorithms (like, e.g., instance based or local models). The main advantage of our method is that it is model independent, i.e., it can be applied with any classifier that provides a probability estimate for a prediction. Moreover, even though we only use it for binary class values, the method can easily be extended to multi-class problems or quantitative prediction (i.e., regression).

A similar approach that computes the most important structural fragment for a single prediction of a query compound has been presented by Ahlberg et al. [44]. Like our approach, this method evaluates the importance of each feature by re-predicting the query compound with a modified feature vector. Our method has binary feature values (a sub-structure does either match or not match a compound), whereas the method by Ahlberg et al. is based on numeric feature values, counting the number of occurrences of a sub-structure. Accordingly, our approach toggles the feature value for estimating the importance of a particular feature, whereas the other method increases the count. Moreover, the method by Ahlberg et al. does not take dependencies between structural features into account (e.g., the count of $N-C$ is increased but not the count for *N*), and absent fragments are not tested.

### Coloring of compound fragments

We depict a circular fingerprint fragment by highlighting the atoms that match this fragment within a compound. Activating fragments are colored in red, deactivating fragments are colored in blue [see *(D1)* and *(D2)* in Fig. 6].

Additionally, we highlight activating and deactivating parts within the query compound. Hence, the weight of each present fragment (i.e., the difference in predicted probability when the feature was swapped) is summed up for all atoms and bonds that match the fragment. The weight is positive for activating and negative for deactivating fragments. Subsequently, the summed-up weights are used as input for a color gradient that ranges from blue (deactivating) to white (neutral) to red (activating).

The implementation of the depiction is based on the CDK and freely available (see “Experimental” section).

## Additional files

**Additional file 1. ** AUPRC and AUROC curves for Table 1.

**Additional file 2 .** Additional validation plots and tables.

## Abbreviations

CoFFer: Collision-free Filtered Circular Fingerprints
(Q)SAR: (quantitative) structure-activity relationship
ECFP: extended-connectivity fingerprint
FCFP: functional class fingerprint
ROC: receiver operating characteristic
AUROC: area under ROC curve
BEDROC: Boltzmann-Enhanced Discrimination of ROC
EF: enrichment factor
AUPRC: area under precision recall curve
CDK: chemistry development kit
REST: representational state transfer
RBF: radial basis function
HPC: high-performance computing

## Footnotes

1 The collision rate of unfiltered fingerprints is 0.025% for 1 million features [4].

2 Currently, only RDKit (http://www.rdkit.org) and CDK [39] provide the computation of ECFP fingerprints (among other types of fingerprints). Open Babel [45] allows calculating MOLPRINT 2D circular fingerprints [46]. The fingerprinting functionalities of the Indigo Toolkit (http://lifescience.opensource.epam.com/indigo) are tree based.

3 In [16], a 50-times repeated holdout validation is performed, using 20% of the compounds for training and 80% as test set. Thus, the models are build on a relatively small amount of the data, which explains the reduced predictivity compared to our approach. Although, the disadvantage of small training datasets was compensated to a degree by increasing the relative amount of active compounds in the training dataset compared to the test dataset.

4 Our applicability domain evaluation is based on the distance measure *D*(*c*), which is defined as the mean Tanimoto distance to the three nearest neighbors in the training dataset (computed with the same structural fragments that are employed by the respective (Q)SAR model). The distance *D*(*c*) is computed for each training compound and the thus created distribution is fitted to a normal distribution. To evaluate a query compound, we compute the cumulative probability $P(X \le x)$ of its distance *D*(*c*), which resembles the probability that a randomly selected training distance is less than or equal to the query compound distance. The query compound is accepted if *P* is less than or equal to 0.95.
