# Supplementary figures and images for: Filtered circular fingerprints improve either prediction or runtime performance while retaining interpretability
Source: J Cheminform. 2016 Oct 31;8:60. doi: 10.1186/s13321-016-0173-z (PMC5088672; doi:10.1186/s13321-016-0173-z)

**Additional file 1 — AUPRC and AUROC curves for Table 1**

AUC

AUPRC

(a)

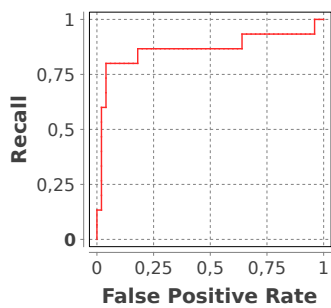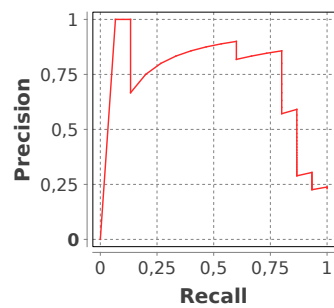

(b)

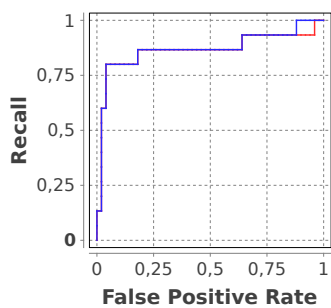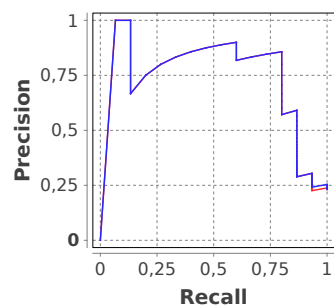

(c)

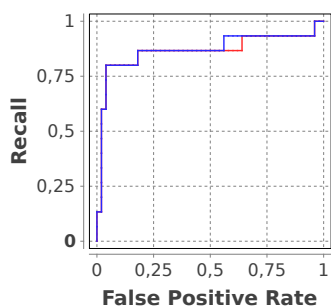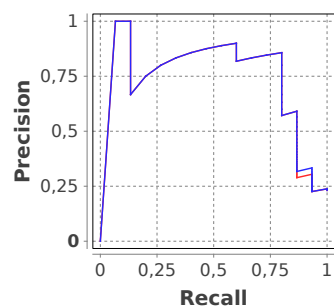

(d)

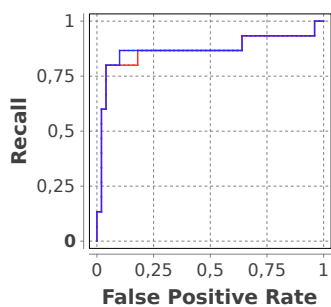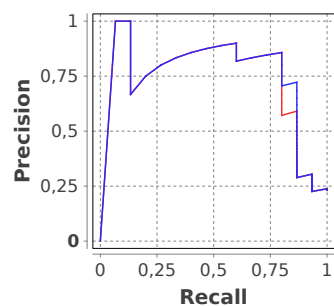

(e)

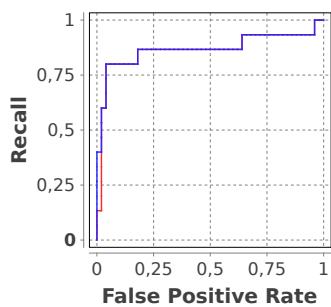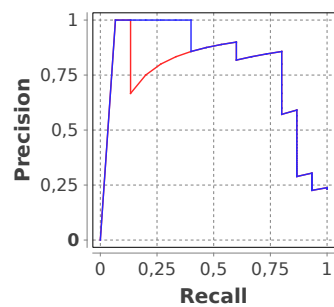

Supplement: Supplementary file 1 — Additional file 1. AUPRC and AUROC curves for Table 1. [file 13321_2016_173_MOESM1_ESM.pdf]
