# Supplementary material for: Filtered circular fingerprints improve either prediction or runtime performance while retaining interpretability
Source: J Cheminform. 2016 Oct 31;8:60. doi: 10.1186/s13321-016-0173-z (PMC5088672; doi:10.1186/s13321-016-0173-z)
Supplement: Supplementary file 2 — Additional file 2 . Additional validation plots and tables. [file 13321_2016_173_MOESM2_ESM.pdf]

This document provides additional information for the article **Filtered Circular Fingerprints Improve Either Prediction Or Runtime Performance While Retaining Interpretability** submitted to the Journal of Cheminformatics by **M. Gütlein and S. Kramer** (contact: guetlein@uni-mainz.de).

Additional file 2 — Additional validation plots and tables

List of Tables

1 Datasets 2
2 Dataset links 3
3 Average number of fragments and bit-collisions with FCFPs. 3
4 Nested cross-validation results 4
5 Comparison to models published for the same datasets 5

List of Figures

1 Support vector machines results for different feature sets (ECFP4, 1024). 6
2 Naive Bayes results for different feature sets (ECFP4, 1024). 7
3 Mean changes in AUROC and AUPRC for different feature sets for 1024. 8
4 Mean changes in AUROC and AUPRC for different feature sets for 4096. 9
5 Mean changes in AUROC and AUPRC for different feature sets for 8192. 10
6 Win loss statistics for ECFP4 with increasing bit-vector sizes for AUROC (instead of AUPRC). 11
7 Win-loss statistics for comparing ECFP diameters for AUROC (instead of AUPRC). 11
8 Win-loss statistics comparing ECFPs vs FCFPs for AUROC (instead of AUPRC). 12

category	name	compounds	active	in-active	target
Balanced	AMES	4337	2401	1936	ames test mutagenicity
Balanced	CPDBAS Mouse	956	430	526	carcinogenicity
Balanced	CPDBAS MultiCellCall	1095	564	531	carcinogenicity
Balanced	CPDBAS Mutagenicity	829	394	435	carcinogenicity
Balanced	CPDBAS Rat	1169	565	604	carcinogenicity
Balanced	CPDBAS SingleCellCall	1464	776	688	carcinogenicity
Balanced	NCTRER	217	126	91	Estrogen receptor
Virtual-Screening	ChEMBL 8	10100	100	10000	tyrosine-protein kinase ABL
Virtual-Screening	ChEMBL 15	10100	100	10000	carbonic anhydrase II
Virtual-Screening	ChEMBL 25	10100	100	10000	glucocorticoid receptor
Virtual-Screening	ChEMBL 36	10100	100	10000	progesterone receptor
Virtual-Screening	ChEMBL 43	10100	100	10000	beta-2 adrenergic receptor
Virtual-Screening	ChEMBL 51	10100	100	10000	serotonin 1a (5-HT1a) receptor
Virtual-Screening	ChEMBL 52	10100	100	10000	alpha-2a adrenergic receptor
Virtual-Screening	ChEMBL 61	10100	100	10000	muscarinic acetylcholine receptor M1
Virtual-Screening	ChEMBL 65	10100	100	10000	cytochrome P450 19A1
Virtual-Screening	ChEMBL 72	10100	100	10000	dopamine D2 receptor
Virtual-Screening	ChEMBL 87	10100	100	10000	cannabinoid CB1 receptor
Virtual-Screening	ChEMBL 90	10100	100	10000	dopamine D4 receptor
Virtual-Screening	ChEMBL 93	10100	100	10000	acetylcholinesterase
Virtual-Screening	ChEMBL 100	10100	100	10000	norepinephrine transporter
Virtual-Screening	ChEMBL 104	10100	100	10000	monoamine oxidase B
Virtual-Screening	ChEMBL 105	10100	100	10000	serotonin 1d (5-HT1d) receptor
Virtual-Screening	ChEMBL 107	10100	100	10000	serotonin 2a (5-HT2a) receptor
Virtual-Screening	ChEMBL 108	10100	100	10000	serotonin 2c (5-HT2c) receptor
Virtual-Screening	ChEMBL 114	10100	100	10000	adenosine A1 receptor
Virtual-Screening	ChEMBL 121	10100	100	10000	serotonin transporter
Virtual-Screening	ChEMBL 126	10100	100	10000	cyclooxygenase-2
Virtual-Screening	ChEMBL 130	10100	100	10000	dopamine D3 receptor
Virtual-Screening	ChEMBL 165	10100	100	10000	HERG
Virtual-Screening	ChEMBL 219	10100	100	10000	muscarinic acetylcholine receptor M3
Virtual-Screening	ChEMBL 259	10100	100	10000	cannabinoid CB2 receptor
Virtual-Screening	ChEMBL 10188	10100	100	10000	MAP kinase p38 alpha
Virtual-Screening	ChEMBL 10193	10100	100	10000	carbonic anhydrase I
Virtual-Screening	ChEMBL 10260	10100	100	10000	vanilloid receptor
Virtual-Screening	ChEMBL 10280	10100	100	10000	histamine H3 receptor
Virtual-Screening	ChEMBL 10378	10100	100	10000	cathepsin B
Virtual-Screening	ChEMBL 10434	10100	100	10000	tyrosine-protein kinase SRC
Virtual-Screening	ChEMBL 10498	10100	100	10000	cathepsin L
Virtual-Screening	ChEMBL 10980	10100	100	10000	vascular endothelial growth factor receptor 2
Virtual-Screening	ChEMBL 11140	10100	100	10000	dipeptidyl peptidase IV
Virtual-Screening	ChEMBL 11359	10100	100	10000	phosphodiesterase 4D
Virtual-Screening	ChEMBL 11365	10100	100	10000	cytochrome P450 2D6
Virtual-Screening	ChEMBL 11489	10100	100	10000	11-beta-hydroxysteroid dehydrogenase 1
Virtual-Screening	ChEMBL 11534	10100	100	10000	cathepsin S
Virtual-Screening	ChEMBL 11575	10100	100	10000	C-C chemokine receptor type 2
Virtual-Screening	ChEMBL 11631	10100	100	10000	sphingosine 1-phosphate receptor Edg-1
Virtual-Screening	ChEMBL 12209	10100	100	10000	carbonic anhydrase XII
Virtual-Screening	ChEMBL 12252	10100	100	10000	beta-secretase 1
Virtual-Screening	ChEMBL 12261	10100	100	10000	c-Jun N-terminal kinase 1
Virtual-Screening	ChEMBL 12670	10100	100	10000	tyrosine-protein kinase receptor FLT3
Virtual-Screening	ChEMBL 12911	10100	100	10000	cytochrome P450 2C9
Virtual-Screening	ChEMBL 12952	10100	100	10000	carbonic anhydrase IX
Virtual-Screening	ChEMBL 13001	10100	100	10000	matrix metalloproteinase-2
Virtual-Screening	ChEMBL 17045	10100	100	10000	cytochrome P450 3A4
Virtual-Screening	ChEMBL 19905	10100	100	10000	melanin-concentrating hormone receptor 1
Virtual-Screening	ChEMBL 100579	10100	100	10000	nicotinic acid receptor 1
Virtual-Screening	DUD cdk2	1779	47	1732	cyclin-dependent kinase
Virtual-Screening	DUD hivrt	1333	31	1302	HIV reverse transcriptase
Virtual-Screening	DUD vegfr2	2355	48	2307	vascular endothelial growth factor receptor
Virtual-Screening	MUV 466	15029	30	14999	S1P1 rec. (GPCR) Agonist
Virtual-Screening	MUV 548	15030	30	15000	PKA (Kinase) Inhibitor
Virtual-Screening	MUV 600	15029	30	14999	SF1 (Nuclear Receptor) Inhibitor
Virtual-Screening	MUV 644	15027	30	14997	Rho-Kinase2 Inhibitor
Virtual-Screening	MUV 652	15030	30	15000	HIV RT-RNase Inhibitor
Virtual-Screening	MUV 689	15023	30	14993	Eph rec. A4 (Rec. Tyr. Kinase) Inhibitor
Virtual-Screening	MUV 692	15030	30	15000	SF1 (Nuclear Receptor) Agonist
Virtual-Screening	MUV 712	15024	30	14994	HSP 90 (Chaperone) Inhibitor
Virtual-Screening	MUV 713	15019	30	14989	ER-a-Coact. Bind. (PPI) Inhibitor
Virtual-Screening	MUV 733	15023	30	14993	ER--Coact. Bind. (PPI) Inhibitor
Virtual-Screening	MUV 737	15026	30	14996	ER-a-Coact. Bind. (PPI) Potentiator
Virtual-Screening	MUV 810	15028	30	14998	FAK (Kinase) Inhibitor
Virtual-Screening	MUV 832	15030	30	15000	Cathepsin G (Protease) Inhibitor
Virtual-Screening	MUV 852	15021	30	14991	FXIIa (Protease) Inhibitor
Virtual-Screening	MUV 858	15030	30	15000	D1 rec. (GPCR) Allosteric Modulator
Virtual-Screening	MUV 859	15029	30	14999	M1 rec. (GPCR) Allosteric Modulator

Table 1 Datasets

dataset/group	source
AMES	http://www.cheminformatics.org/datasets/bursi
CPDBAS	http://www.epa.gov/ncct/dsstox/sdf_cpdbas.html
NCTRER	http://www.epa.gov/ncct/dsstox/sdf_nctrer.html
ChEMBL	https://github.com/rdkit/benchmarking_platform
DUD	http://dud.docking.org, https://github.com/rdkit/benchmarking_platform
MUV	http://www.pharmchem.tu-bs.de/lehre/baumann/MUV.html, https://github.com/rdkit/benchmarking_platform

Table 2 Dataset links

		1024		2048		4096		8192	
Type	Fragments	rate	bit-load	rate	bit-load	rate	bit-load	rate	bit-load
fcfp6	41120.71	0.99	40.16	0.98	20.1	0.96	10.08	0.91	5.11
fcfp4	7497.14	0.97	7.35	0.88	3.78	0.64	2.19		
fcfp2	325.47								
fcfp0	13.5								

Table 3 Average number of fragments and bit-collisions with FCFPs.

Rate is the ratio of bit positions that are mapped by more than one fragment (e.g., 97% of bit-positions correspond to multiple fragments for FCFP4 and bit-vector size 1024). *Bit-load* is the mean number of fragments that are mapped to a single bit position.

Dataset	Algorithm	AlgParams	#Frags	Accur.	AUC	AUPRC	Sensit.	Specif.
AMES	RF		4096	0.837	0.91	0.922	0.849	0.822
CPDBAS Mouse	NB		2048	0.655	0.72	0.684	0.664	0.648
CPDBAS MultiCellCall	RF		2048	0.692	0.767	0.794	0.716	0.667
CPDBAS Mutagenicity	RF		1024	0.772	0.837	0.84	0.723	0.816
CPDBAS Rat	RF		2048	0.664	0.717	0.712	0.622	0.703
CPDBAS SingleCellCall	RF		2048	0.672	0.733	0.767	0.714	0.625
DUD cdk2	SVM	C:1 Linear	2048	0.993	0.99	0.923	0.755	0.999
DUD hivrt	SVM	C:100 RBF Gamma:0.01	2048	0.988	0.986	0.859	0.483	1
DUD vegfr2	SVM	C:10 RBF Gamma:0.01	2048	0.993	0.998	0.958	0.687	1
NCTRER	RF		1024	0.871	0.931	0.952	0.884	0.853
ChEMBL 100	SVM	C:100 RBF Gamma:0.001	2048	0.995	0.963	0.731	0.56	0.999
ChEMBL 100579	RF		1024	0.998	1	0.999	0.84	1
ChEMBL 10188	SVM	C:10 RBF Gamma:0.01	4096	0.995	0.97	0.805	0.68	0.998
ChEMBL 10193	SVM	C:10 RBF Gamma:0.01	8192	0.996	0.986	0.84	0.663	0.999
ChEMBL 10260	SVM	C:10 RBF Gamma:0.01	8192	0.994	0.967	0.828	0.45	1
ChEMBL 10280	SVM	C:10 RBF Gamma:0.01	1024	0.996	0.995	0.927	0.627	1
ChEMBL 10378	SVM	C:100 RBF Gamma:0.001	1024	0.996	0.992	0.942	0.65	1
ChEMBL 104	SVM	C:100 RBF Gamma:0.01	8192	0.995	0.986	0.821	0.547	0.999
ChEMBL 10434	SVM	C:10 RBF Gamma:0.01	2048	0.996	0.973	0.829	0.687	0.999
ChEMBL 10498	RF		1024	0.995	0.992	0.931	0.537	1
ChEMBL 105	SVM	C:100 RBF Gamma:0.001	1024	0.999	0.999	0.98	0.877	1
ChEMBL 107	SVM	C:100 RBF Gamma:0.001	2048	0.995	0.98	0.766	0.637	0.999
ChEMBL 108	SVM	C:10 RBF Gamma:0.01	8192	0.994	0.975	0.689	0.503	0.999
ChEMBL 10980	SVM	C:10 RBF Gamma:0.01	8192	0.994	0.986	0.826	0.42	1
ChEMBL 11140	SVM	C:10 RBF Gamma:0.01	1024	0.997	0.998	0.955	0.74	1
ChEMBL 11359	SVM	C:100 RBF Gamma:0.001	1024	0.998	0.983	0.956	0.823	1
ChEMBL 11365	SVM	C:10 RBF Gamma:0.01	8192	0.992	0.95	0.501	0.367	0.998
ChEMBL 114	RF		2048	0.993	0.967	0.693	0.367	0.999
ChEMBL 11489	SVM	C:10 RBF Gamma:0.01	8192	0.992	0.94	0.601	0.48	0.997
ChEMBL 11534	RF		1024	0.996	0.988	0.925	0.6	1
ChEMBL 11575	SVM	C:10 RBF Gamma:0.01	1024	0.997	0.968	0.89	0.817	0.999
ChEMBL 11631	SVM	C:10 RBF Gamma:0.01	4096	0.997	0.979	0.872	0.73	1
ChEMBL 121	SVM	C:10 RBF Gamma:0.01	4096	0.995	0.987	0.809	0.62	0.999
ChEMBL 12209	SVM	C:10 RBF Gamma:0.01	2048	0.996	0.981	0.862	0.77	0.998
ChEMBL 12252	SVM	C:10 RBF Gamma:0.01	8192	0.996	0.974	0.87	0.643	1
ChEMBL 12261	SVM	C:10 RBF Gamma:0.01	4096	0.997	0.989	0.888	0.697	1
ChEMBL 126	SVM	C:10 RBF Gamma:0.01	8192	0.993	0.977	0.808	0.477	0.998
ChEMBL 12670	SVM	C:10 RBF Gamma:0.01	8192	0.996	0.99	0.877	0.63	1
ChEMBL 12911	SVM	C:10 RBF Gamma:0.01	8192	0.988	0.91	0.392	0.37	0.994
ChEMBL 12952	RF		2048	0.995	0.993	0.851	0.64	0.999
ChEMBL 130	SVM	C:10 RBF Gamma:0.01	8192	0.994	0.98	0.783	0.427	1
ChEMBL 13001	RF		1024	0.995	0.976	0.882	0.527	1
ChEMBL 15	SVM	C:10 RBF Gamma:0.01	1024	0.994	0.981	0.815	0.56	0.998
ChEMBL 165	SVM	C:100 RBF Gamma:0.001	8192	0.992	0.947	0.573	0.453	0.998
ChEMBL 17045	SVM	C:10 RBF Gamma:0.001	8192	0.987	0.91	0.455	0.483	0.992
ChEMBL 19905	SVM	C:100 RBF Gamma:0.01	4096	0.996	0.985	0.872	0.6	1
ChEMBL 219	SVM	C:10 RBF Gamma:0.01	8192	0.996	0.987	0.861	0.643	1
ChEMBL 25	SVM	C:100 RBF Gamma:0.001	4096	0.998	0.99	0.914	0.84	0.999
ChEMBL 259	SVM	C:10 RBF Gamma:0.01	8192	0.994	0.954	0.739	0.457	1
ChEMBL 36	RF		1024	0.998	0.999	0.972	0.753	1
ChEMBL 43	RF		2048	0.992	0.856	0.501	0.383	0.998
ChEMBL 51	SVM	C:100 RBF Gamma:0.01	8192	0.996	0.986	0.903	0.583	1
ChEMBL 52	SVM	C:10 RBF Gamma:0.01	8192	0.994	0.969	0.742	0.547	0.999
ChEMBL 61	SVM	C:100 RBF Gamma:0.01	8192	0.994	0.969	0.728	0.467	1
ChEMBL 65	SVM	C:10 RBF Gamma:0.01	8192	0.995	0.99	0.834	0.593	0.999
ChEMBL 72	SVM	C:10 RBF Gamma:0.01	8192	0.996	0.99	0.87	0.603	1
ChEMBL 8	SVM	C:100 RBF Gamma:0.001	2048	0.996	0.985	0.829	0.673	0.999
ChEMBL 87	SVM	C:10 RBF Gamma:0.01	8192	0.993	0.932	0.619	0.277	1
ChEMBL 90	SVM	C:10 RBF Gamma:0.01	8192	0.996	0.995	0.872	0.717	0.999
ChEMBL 93	SVM	C:100 RBF Gamma:0.001	2048	0.995	0.983	0.826	0.763	0.998
MUV 466	SVM	C:10 Linear	1024	0.997	0.688	0.065	0.044	0.999
MUV 548	SVM	C:10 RBF Gamma:0.01	4096	0.997	0.86	0.227	0.122	0.999
MUV 600	SVM	C:10 RBF Gamma:0.1	1024	0.997	0.726	0.073	0.033	0.999
MUV 644	SVM	C:100 RBF Gamma:0.01	2048	0.998	0.885	0.205	0.033	1
MUV 652	SVM	C:100 RBF Gamma:0.01	8192	0.998	0.712	0.086	0.011	1
MUV 689	SVM	C:10 Linear	1024	0.997	0.797	0.112	0.056	0.999
MUV 692	SVM	C:100 RBF Gamma:0.001	1024	0.996	0.544	0.008	0	0.998
MUV 712	SVM	C:100 RBF Gamma:0.01	4096	0.997	0.83	0.199	0.156	0.998
MUV 713	SVM	C:100 RBF Gamma:0.01	1024	0.997	0.689	0.068	0.067	0.999
MUV 733	SVM	C:1 RBF Gamma:0.01	1024	0.995	0.608	0.102	0.089	0.997
MUV 737	SVM	C:100 RBF Gamma:0.01	4096	0.996	0.67	0.052	0.033	0.998
MUV 810	SVM	C:100 RBF Gamma:0.01	8192	0.998	0.794	0.161	0.067	1
MUV 832	SVM	C:100 RBF Gamma:0.01	1024	0.998	0.93	0.556	0.389	0.999
MUV 852	SVM	C:1 RBF Gamma:0.01	1024	0.998	0.84	0.397	0.356	0.999
MUV 858	NB		1024	0.996	0.688	0.149	0.144	0.997
MUV 859	SVM	C:1 Linear	1024	0.997	0.56	0.008	0	0.999

Table 4 Nested cross-validation results

dataset	measure	this work	other approach	id
AMES	AUROC	**0.910**	0.835	a)
	AUROC	**0.910**	0.909	b)
CPDB Mutagenicity	AUROC	**0.834**	0.786	a)
CPDB Rat	Accuracy	**66.4**	61.4	c)
NCTRER	AUROC	**0.931**	0.806	d)
	Accuracy	**0.871**	0.857	e)
MUV 466	AUROC	**0,688**	0,663	b)
MUV 548	AUROC	0,860	**0,881**	b)
MUV 600	AUROC	**0,726**	0,673	b)
MUV 644	AUROC	0,885	**0,895**	b)
MUV 652	AUROC	0,712	**0,810**	b)
MUV 689	AUROC	**0,797**	0,730	b)
MUV 692	AUROC	0,544	**0,589**	b)
MUV 712	AUROC	**0,830**	0,813	b)
MUV 713	AUROC	0,689	**0,703**	b)
MUV 733	AUROC	0,608	**0,666**	b)
MUV 737	AUROC	0,670	**0,671**	b)
MUV 810	AUROC	**0,794**	0,773	b)
MUV 832	AUROC	**0,930**	0,921	b)
MUV 852	AUROC	**0,840**	0,821	b)
MUV 858	AUROC	0,688	0,688	b)
MUV 859	AUROC	0,560	**0,602**	b)

approach id	validation scheme	additional info
a)	LOO-CV	http://lazar.in-silico.de (lazar skips some compounds from prediction)
b)	nested 5-fold CV	high level of SVM optimization
c)	test set validation	curated version of the dataset
d)	10 × 10-fold CV	
e)	holdout 33% test data	

approach id	author	year	publication
a)	Helma	2006	https://dx.doi.org/10.1007/s11030-005-9001-5
b)	Rosenbaum	2011	https://dx.doi.org/10.1186/1758-2946-3-11
c)	Fjodorova	2010	https://dx.doi.org/10.1186/1752-153X-4-S1-S3
d)	Karwath	2006	https://dx.doi.org/10.1021/ci060159g
e)	Cao	2012	https://dx.doi.org/10.1002/cem.1416

Table 5 Comparison to models published for the same datasets

As noted in the article a fair comparison to Riniker et. al (2013, https://dx.doi.org/10.1021/ci400466r) is not possible, our models have a higher AUROC in 66/69 cases, their results can be found in supplementary file ci400466r_si_007.zip in table figure5_datasetsI_auc.csv here: http://pubs.acs.org/doi/suppl/10.1021/ci400466r

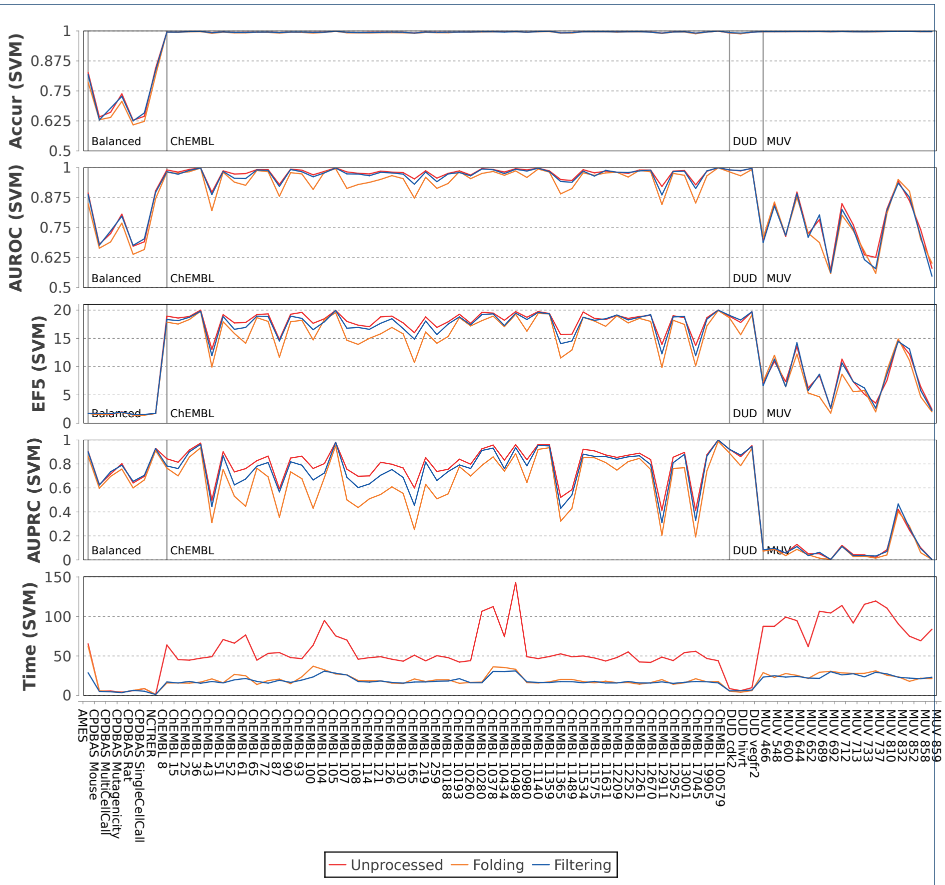

Figure 1 Support vector machines results for different feature sets (ECFP4, 1024).

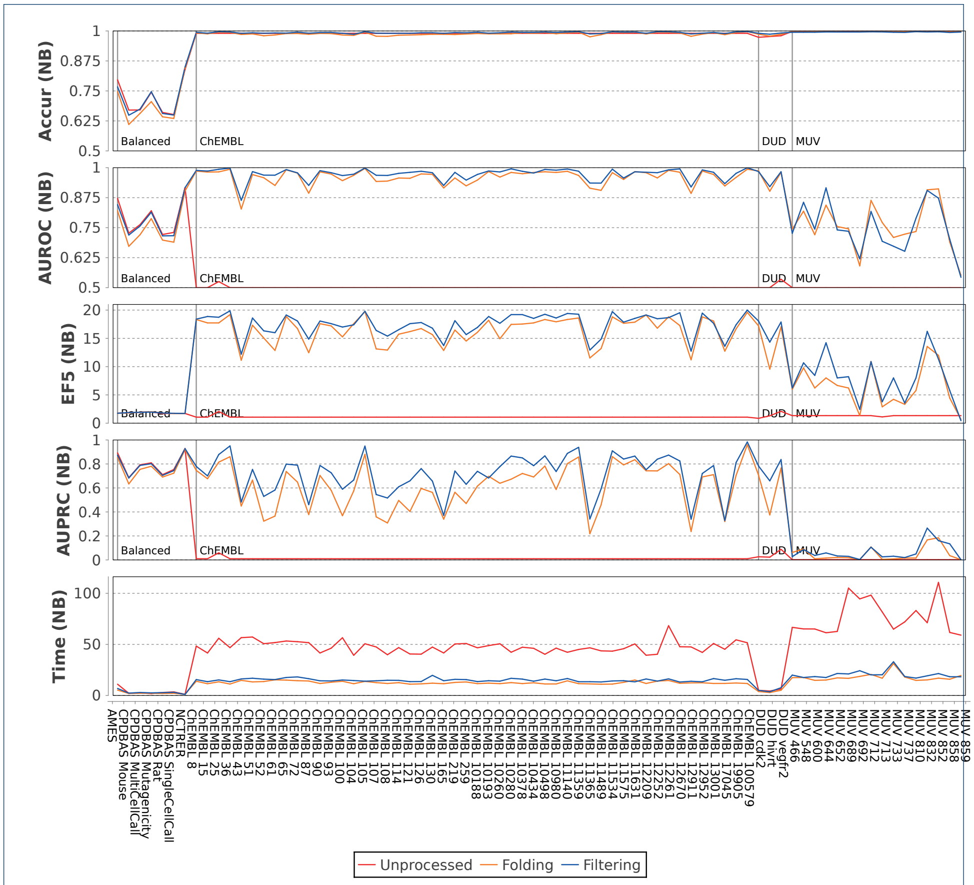

Figure 2 Naive Bayes results for different feature sets (ECFP4, 1024).

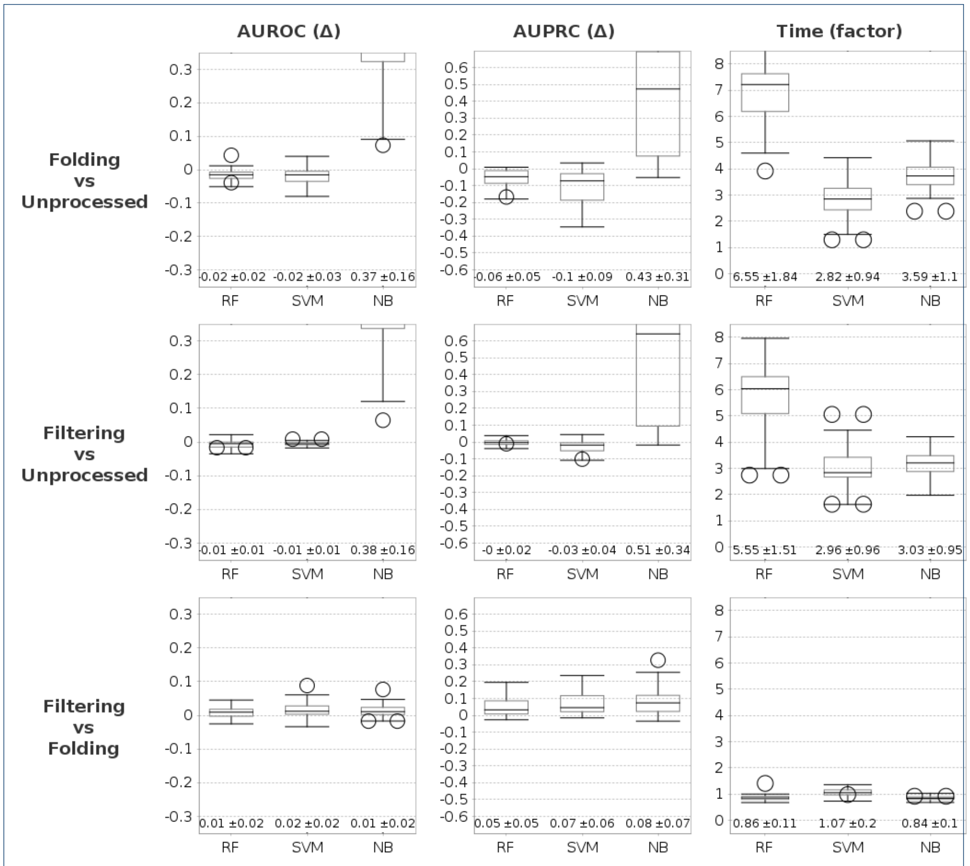

Figure 3 Mean changes in AUROC and AUPRC for different feature sets for 1024.

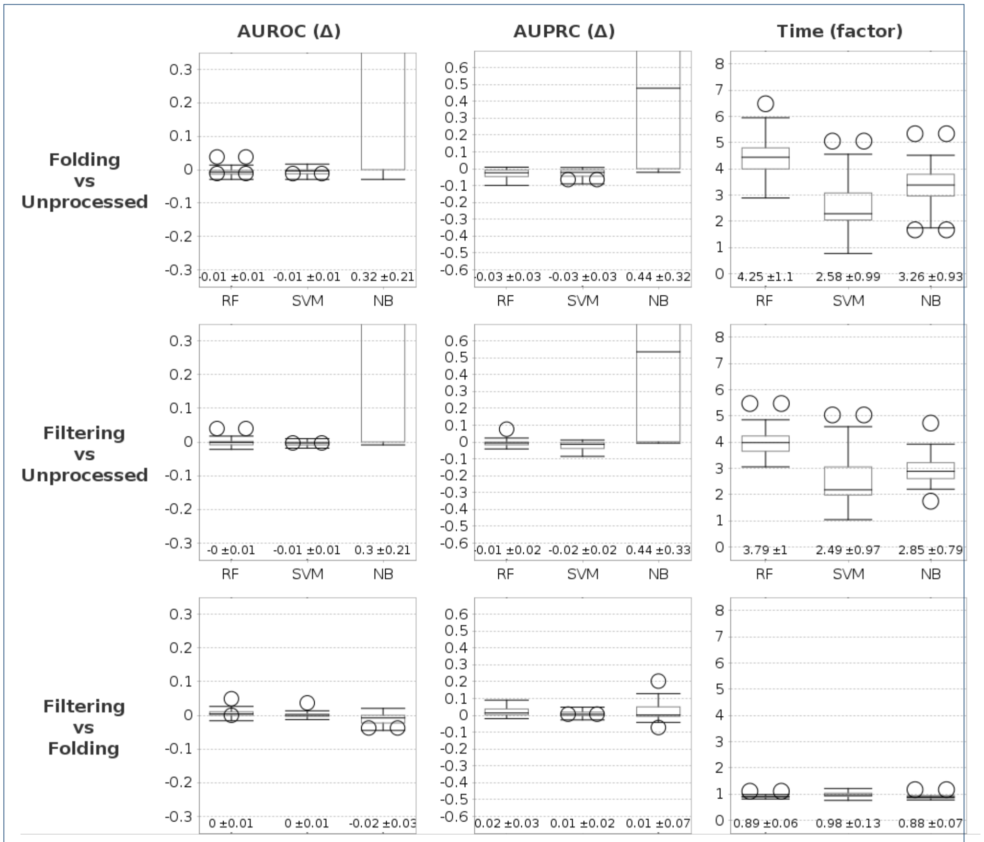

Figure 4 Mean changes in AUROC and AUPRC for different feature sets for 4096.

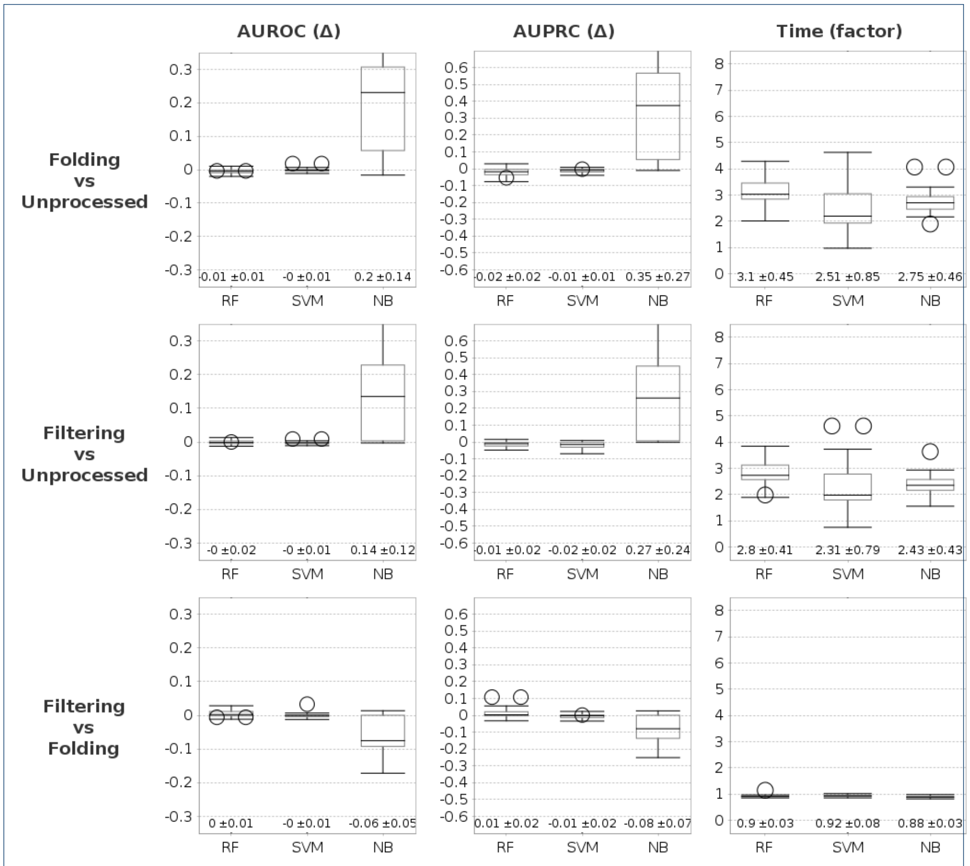

Figure 5 Mean changes in AUROC and AUPRC for different feature sets for 8192.

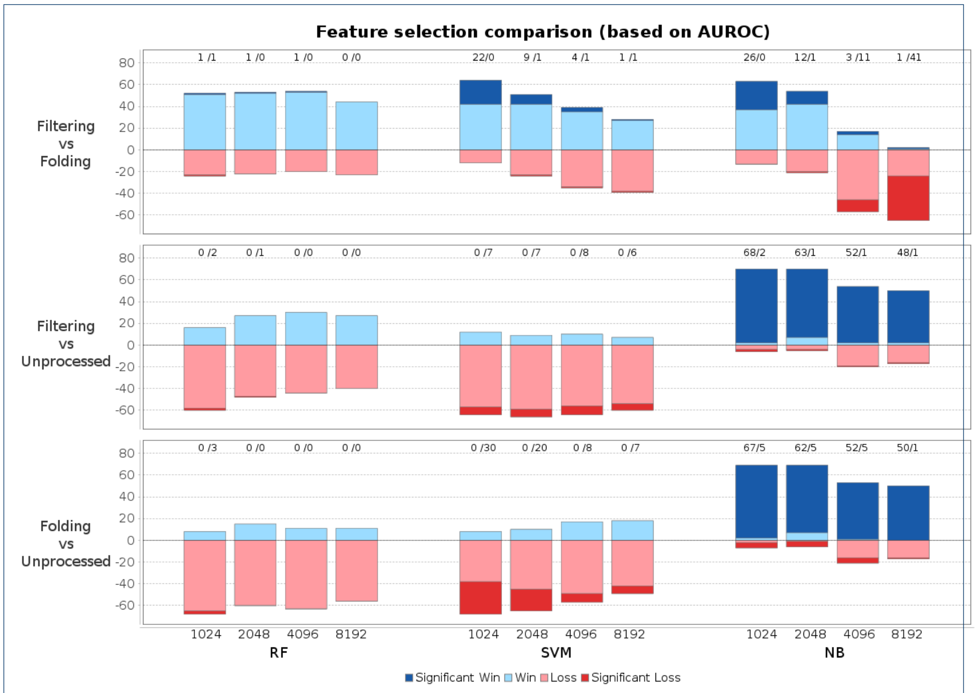

Figure 6 Win loss statistics for ECFP4 with increasing bit-vector sizes for AUROC (instead of AUPRC).

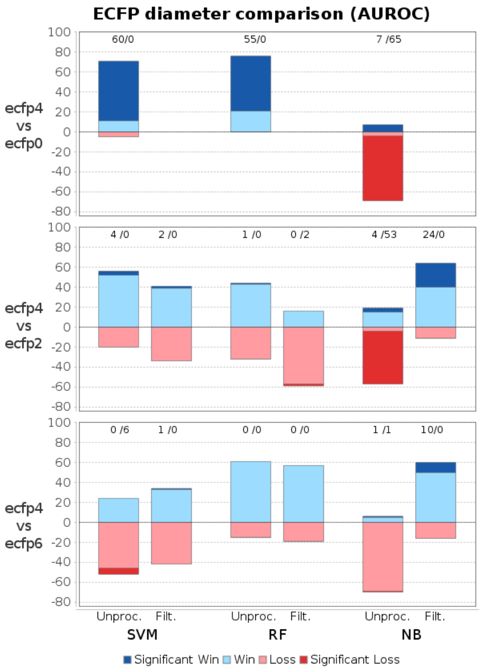

Figure 7 Win-loss statistics for comparing ECFP diameters for AUROC (instead of AUPRC).

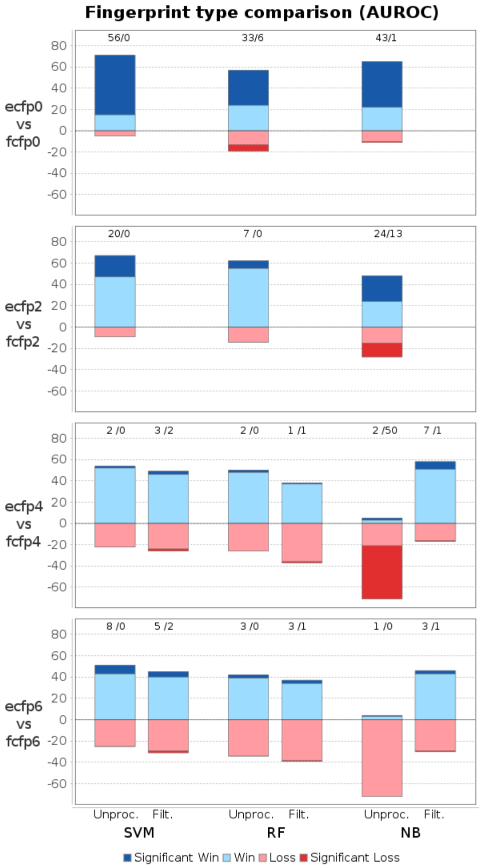

Figure 8 Win-loss statistics comparing ECFPs vs FCFPs for AUROC (instead of AUPRC).
